# Predicting fiber content in herbivore fecal samples using a multispecies NIRS model

**DOI:** 10.1371/journal.pone.0317145

**Published:** 2025-01-08

**Authors:** Mariana Rossa, Emmanuel Serrano, João Carvalho, Néstor Fernández, Jorge R. López-Olvera, Mathieu Garel, João P. V. Santos, Maurizio Ramanzin, Pia Anderwald, Pierangelo Freschi, Jordi Bartolomé, Santiago Lavín, Elena Albanell

**Affiliations:** 1 Departamento de Biologia e Centro de Estudos do Ambiente e do Mar (CESAM), Universidade de Aveiro, Campus Universitário de Santiago, Aveiro, Portugal; 2 Wildlife Ecology & Health Group (WE&H) and Servei d’Ecopatologia de Fauna Salvatge (SEFaS), Departament de Medicina i Cirurgia Animals, Facultat de Veterinària, Universitat Autònoma de Barcelona (UAB), Bellaterra, Barcelona, Spain; 3 Department of Conservation Biology, Estación Biológica de Doñana, Consejo Superior de Investigaciones Científicas EBD-CSIC, Sevilla, Spain; 4 German Centre for Integrative Biodiversity Research (iDiv) Halle-Jena-Leipzig, Leipzig, Germany; 5 Institute of Biology, Martin Luther University Halle-Wittenberg, Halle, Germany; 6 Office Français de la Biodiversité (OFB–Service Anthropisation et Fonctionnement des Ecosystèmes Terrestres), Gières, France; 7 Palombar–Associação de Conservação da Natureza e do Patrimonio Rural, Antiga Escola Primária, Uva, Vimioso, Portugal; 8 CIBIO–Centro de Investigação em Biodiversidade e Recursos Genéticos, InBIO Laboratório Associado, Campus de Vairão, Universidade do Porto, Vairão, Portugal; 9 BIOPOLIS Program in Genomics, Biodiversity and Land Planning, CIBIO, Campus de Vairão, Vairão, Portugal; 10 Department of Agronomy, Food, Natural Resources, Animals and Environment (DAFNAE), University of Padova (UNIPD), Agripolis, Legnaro, Italy; 11 Swiss National Park, Chastè Planta-Wildenberg, Zernez, Switzerland; 12 School of Agricultural, Forestry, Food and Environmental Sciences (SAFE), University of Basilicata, Potenza, Italy; 13 Group of Research in Ruminants (G2R), Department of Animal and Food Sciences, Universitat Autònoma de Barcelona, Bellaterra, Spain; University of Agriculture Faisalabad, PAKISTAN

## Abstract

Fiber is essential for rumen health, microbial fermentation, and the energy supply of herbivores. Even though the study of fecal fiber contents (neutral detergent fiber NDF, acid detergent fiber ADF, and acid detergent lignin ADL) using near-infrared reflectance spectroscopy (NIRS) has allowed investigating nutritional ecology of different herbivore species, NIRS calibrations are species-specific and require a large number of samples for predictions. A multispecies calibration would be an advantage since samples from different herbivores could be used to calibrate a model capable of predicting the fecal fiber content of other herbivores. To date, however, multispecies models have not been developed to predict fiber contents in the feces of herbivores. Here, we fill this gap by calibrating three fiber multispecies models (NDF, ADF and ADL) using fecal samples from domestic and wild herbivore species. We also evaluated the effect of incorporating sodium sulfite in fiber determination protocol. The initial dataset consisting of 445 samples of six herbivore species was used to calibrate (80% of the samples) and validate (20% of the samples) the models. Subsequently, 63 samples of five herbivores not included in the calibration set were used for the external validation of the model. Since sodium sulfite did not significantly improve fecal fiber prediction, our model was developed without this compound. The multispecies models obtained were highly accurate determining NDF, ADF and ADL (R^2^_CAL_, coefficient of determination in calibration, ≥ 0.93, R^2^_VAL_, coefficient of determination in validation, ≥ 0.91) and independent of external confounders. For external validation, the accuracy in predicting fecal samples in other herbivore species was also satisfactory, with consistently better values for NDF (R^2^_VAL_, 0.86–0.94) and ADF (R^2^_VAL_, 0.80–0.95) than for ADL (R^2^_VAL_, 0.66–0.89). We show that multispecies NIRS calibrations can be used with high accuracy to assess fecal fiber contents across diverse herbivore species. This finding represents a significant advance in the study of the nutritional ecology of herbivores with contrasting foraging patterns. In the future, widening the data range (*e*.*g*., species and locations) of the initial dataset could further improve the accuracy of these models.

## Introduction

Nutritional ecology of herbivores provides insights into the relationship between the natural history of animals and their environment [1]. The study of nutritional ecology relies on different tools to understand how dietary patterns, nutritional requirements, and foraging strategies of herbivores are influenced by factors such as quality and availability of food [2, 3], the health status of individuals [4], and changes in environmental conditions [5, 6]. Understanding the nutritional ecology of herbivores is essential for effective wildlife management, conservation, and maintaining functional ecosystems. Continuous monitoring of nutritional quality of diet requires informative and readily available samples. Fecal samples can be easily and non-invasively collected during field surveys. This has significantly contributed to the use of fecal material analysis to infer the diet composition and quality of herbivores living in contrasting environments such as deserts [7] or high mountains [8].

Although all herbivores feed on plant matter, some variability can be found in their diet and physiology. Based on their diet, herbivores can be considered browsers, grazers or intermediate feeders. The former feed mainly on woody species and herbs, and the latter on grass [9]. Intermediate feeders are all animals that can change their diet depending on the availability of plants in the environment. In terms of physiology, foregut fermenters or ruminant fermenters are those who ingest moderate amounts of fiber, have large rumens where they digest fiber, have a longer retention time and a higher fiber digestibility, and hindgut fermenters, having small rumens, consume a greater amount of food, have a shorter retention time and a lower fiber digestibility, which occurs mainly in the caecum [10]. Furthermore, some herbivores have characteristics for increasing fiber absorption, such as coprophagous mammals that produce soft feces (rich in water, fiber and enzymes) that will be ingested and digested again, producing hard feces (low content of water and fiber; [11]). Thus, it is expected that the amount of fiber digested and excreted varies between species due to a combination of their diet and physiology.

Essential to the diet of herbivores, fiber content is related to the proportion of components resistant to herbivore digestive enzymes found in feces, such as cellulose, hemicellulose, and lignin. Fecal fiber proportion can thus serve as indicator of diet quality for herbivores by reflecting diet composition [12] and forage quality [13] of herbivores. For instance, high fecal fiber contents may suggest that herbivores are consuming diets with a significant amount of indigestible material [14], and, consequently, low nutritional value. Thus, high fecal fiber content can be due to factors like low-quality forage and/or high fiber content in the available vegetation [15]. This link between diet and fecal fiber is supported by experimental studies comparing the amount of digested and excreted fiber in diets with contrasting digestibility [16–18]. This positive correlation between ingested and excreted fiber [19] enables the detection of changes in forage quality through fecal fiber analysis [2]. There is a wide variety of applications for the study of fiber in diets, and the information generated is relevant to the management and conservation of herbivore populations. For example, fecal fiber analysis has been used to assess density-dependent effects on herbivore nutrition [20, 21].

The most common fiber content determination procedure for the analysis of animal feeds was developed by Van Soest and colleagues [14] and has been later repeatedly modified to improve the procedure (*e*.*g*., [22]). Fibers are analyzed sequentially as three separate fractions related to the composition of plant cell walls [14], namely neutral detergent fiber (NDF), acid detergent fiber (ADF), and acid detergent lignin (ADL). Sodium sulfite can be used optionally for neutral detergent fiber determination to increase the solubilization of proteins, since it cleaves disulfide linkages in proteins bound to the cell wall [14], allowing the removal of protein contamination in fiber content determination [23]. However, sodium sulfite also solubilizes fibrous compounds, such as lignin, and consequently, NDF, ADF, and ADL content values are lower when sodium sulfite is used in the sequential analysis [24]. Therefore, sodium sulfite is not recommended in fiber sequential analyses [14]. However, there are advantages and disadvantages in both procedures which result in different NDF values. Therefore, a preliminary study is necessary to determine the best fiber content determination protocol.

Near-infrared reflectance spectroscopy (NIRS), together with some machine-learning processes [25], can create a calibration model using the laboratory chemical analysis values and absorption spectra of the same samples. This model is trained with an initial dataset of samples and subsequently validated with samples not used for the calibration. Once calibrated and validated, the model allows the rapid prediction of values for future samples without the need for laboratory chemical analysis [26]. NIRS prediction models allow large amounts of information to be predicted quickly and easily without destroying samples or using reagents [27]. The sample types used with this methodology can include stomach contents, feces, forage, and feeds [18, 28–32]. Fecal samples (fNIRS) are frequently used in nutritional ecology (*e*.*g*., [33–37]) due to the ease of collection and high amount of information obtained. Multiple studies have used fNIRS in herbivores for nutritional ecology studies by assessing fecal fiber (*e*.*g*., [38]), fecal nitrogen (*e*.*g*., [39]) and fecal glucocorticoid metabolites as a proxy for stress (*e*.*g*., [40]).

Most of the fNIRS fiber content studies are species- and population-specific (*e*.*g*., [12, 41]). In this context, calibration procedures typically necessitate a minimum of 50 independent samples for small, homogeneous populations, or a minimum of 150 samples for large, more diverse populations [42]. However, for endangered species and/or small populations, it is laborious to collect a sufficient number of fecal samples for calibration. Thus, efforts have been made to overcome the constraints of limited sample availability (*e*.*g*., [43, 44]). Multispecies models are increasingly being developed for calibrations for multiple forages and feeds [45–47]. These multispecies models allow calibration with cross-species samples (thus requiring fewer samples per species) to extrapolate values for different species. This is possible when the chemical compositions and spectral information overlap widely between species. In diet quality analysis using fNIRS, accurate and robust multispecies calibrations models for fecal nitrogen [36] and fecal phosphorus [48] have also been recently developed. However, to the best of our knowledge, there are no multispecies calibrations for fiber contents.

In this study, our aim was to develop, calibrate, and validate three NIRS multispecies models to predict fecal fiber content, namely NDF, ADF and ADL contents. Since the fiber determination can include the optional use of sodium sulfite during laboratory analysis [14], we performed two pre-calibrations to compare the results with and without the addition of sodium sulfite. Then, we developed the fiber multispecies models by using fecal samples of six herbivores with contrasting digestive physiologies and diet preference. Finally, we tested if the obtained fiber multispecies models could be applied to predict the fecal fiber content from herbivore species not included in the original multispecies calibration.

## Materials and methods

### Ethics statement

During the study, no endangered or protected species were sampled, so specific permissions were not required. All the fecal samples were collected from the ground, except for those of the red deer, roe deer and mouflon, which were taken from hunted individuals. These three ungulate species were legally hunted in their own habitat by authorized gamekeepers and hunters within the framework of scientific programs approved by the competent authorities (*e*.*g*., French Ministry of Environment for roe deer and mouflon) or annual hunting plans approved by France, Portugal and Spain.

### Fecal samples

**Fig 1:**
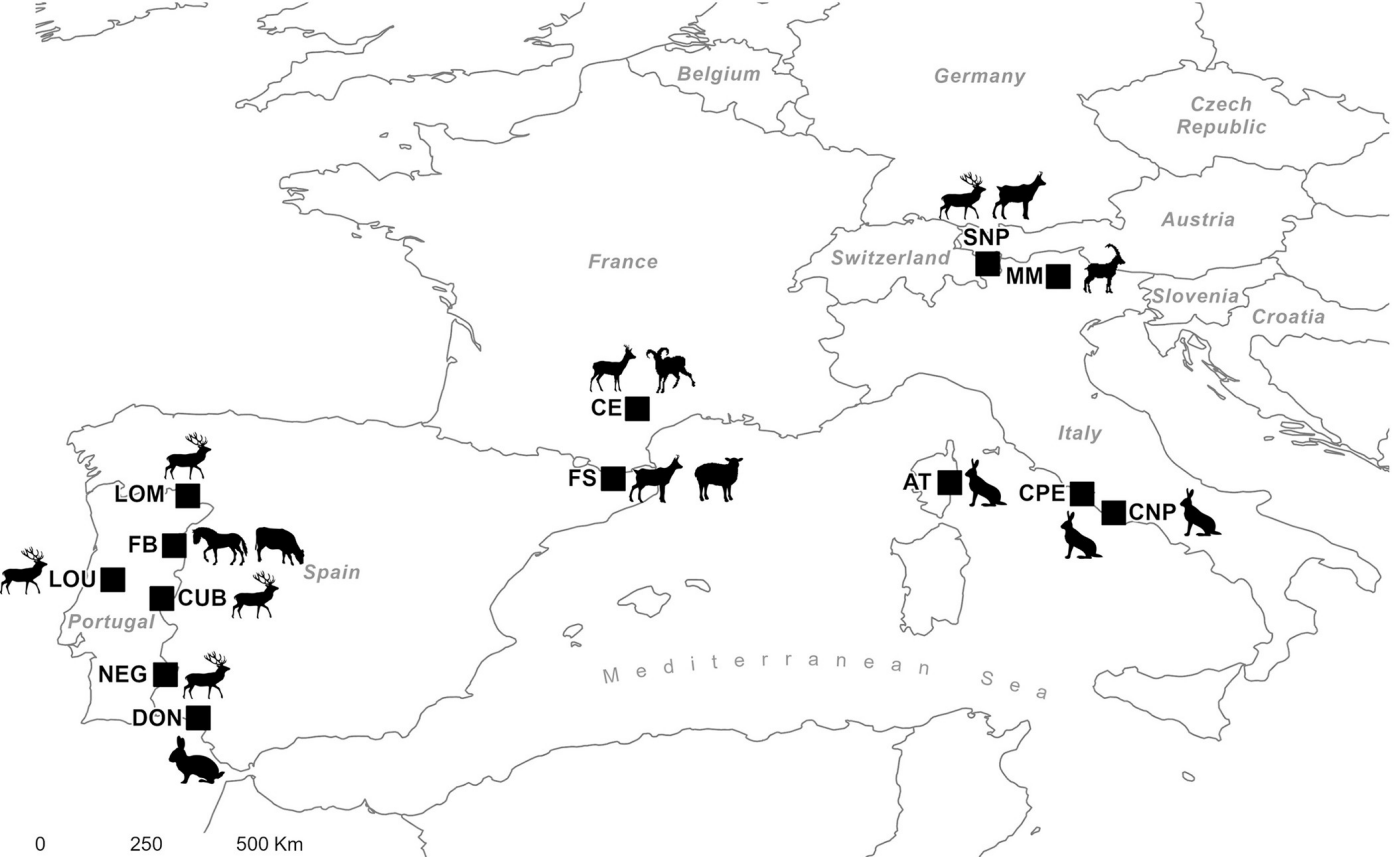
Map showing the geographical distribution of fecal samples by herbivore species.

Between 2016 and 2022, a total of 508 fresh fecal samples of eleven herbivore species with different digestive physiologies and diet preferences (*i*.*e*., grazer, browser and intermediate), were collected from different geographic areas (Table 1 and Fig 1) covering all seasons to account for variations in the diets. This broad sample selection focused on the importance of spectral diversity. All samples were kept frozen at -20°C until analysis.

**Table 1:** Herbivore species, origin and the number (N) of fecal samples used in the study.

Species	Country	N	Location
Cattle ^b^^,^ ^d^ (*Bos taurus*)	Portugal	11	Faia Brava Reserve, North-Eastern Portugal
Alpine ibex ^b^^,^ ^e^ (*Capra ibex*)	Italy	100	Marmolada massif, Eastern Italian Alps
Roe deer ^b^^, c^^a3^ (*Capreolus capreolus*)	France	11	Caroux-Espinouse massif, Southern France
Red deer ^b^^,^ ^e^ (*Cervus elaphus*)	Portugal	76	Lombada National Hunting Area (North-Eastern Portugal), Lousã Mountain (Central Portugal), Cubeira Tourist Hunting Area (Central Portugal) and Herdade da Negrita Tourist Hunting Area (South-Eastern Portugal)
	Switzerland	24	Swiss National Park, South-Eastern Switzerland
Horse ^a^^,^ ^d^ (*Equus caballus*)	Portugal	11	Faia Brava Reserve, North-Eastern Portugal
Italian hare ^a^ (*Lepus corsicanus*)	Italy	54	Circeo National Park and Castelporziano Presidential Estate, Latium, Central Italy
	France	18	Aleria and Tallone districts, Corsica
European rabbit ^a^ (*Oryctolagus cuniculus*)	Spain	10	Doñana National Park, South-Western Spain
Sheep ^b^^,^ ^d^ (*Ovis aries*)	Spain	100	Freser-Setcases National Game Reserve, Eastern Pyrenees
European mouflon ^b^^,^ ^e^ (*Ovis gmelini musimon*)	France	20	Caroux-Espinouse massif, Southern France
Pyrenean chamois ^b^^,^ ^c^ (*Rupicapra pyrenaica*)	Spain	48	Freser-Setcases National Game Reserve, Eastern Pyrenees
Alpine chamois ^b^^,^ ^c^ (*Rupicapra rupicapra*)	Switzerland	30	Swiss National Park, South-Eastern Switzerland

^a^ Hindgut fermenter

^b^ Foregut fermenter

^c^ Browser

^d^ Grazer

^e^ Intermediate feeders.

### Chemical analysis

Frozen feces were thawed and lyophilized or oven-dried at 60°C for 24h to constant weight. Subsequently, samples were ground using a laboratory mill equipped with a 1 mm sieve (Cyclotec 1093, FOSS Tecator, Höganäs, Sweden). Dry matter of each sample was determined in duplicate after using a drying oven at 103°C for 24 h. To determine fiber contents, the samples were analyzed sequentially for neutral detergent fiber (NDF), acid detergent fiber (ADF), and acid detergent lignin (ADL) as described following the Van Soest method [14], using an Ankom 200 Fibre Analyser incubator (Ankom Technology, Macedon, NY, USA). Fiber analysis was determined on an ash-free basis and without alpha amylase. All analyses were carried out in duplicate and the coefficient of variation for each fiber analysis was 0.99% (NDF), 1.21% (ADF) and 3.18% (ADL). During pre-calibrations, samples were also treated with and without the use of sodium sulfite to define the best protocol option for the multispecies models. The results were expressed as a percentage (%) of dry matter (DM). The differences in NDF, ADF and ADL values between samples treated or not with sodium sulfite in the NDF analysis were assessed with a Paired T-Test, and Pearson correlations among traits were also calculated using the CORR procedure in SAS ver. 9.4 (SAS Institute Inc., Cary, NC).

### NIRS analysis and calibration procedure

All the ground fecal samples were packed in closed ring cup cells containing 2–3 g of the sample and scanned using a NIRSystems 5000 scanning monochromator (FOSS, Hillerød, Denmark). The NIR spectra were recorded in the reflectance mode at 2 nm intervals from 1108 to 2492 nm which gave 692 data points for each sample, according to our previously described procedure [49, 50].

WinISI 4.10 (Infrasoft International, Port Matilda, PA, USA) software was used for data processing and development of chemometric models. The spectral preprocessing methods employed to remove slope variation, correct scatter effects and reduce the effects of particle size were the standard normal variate (SNV), detrend (D) and the multiplicative scatter correction (MSC) [51]. The fecal fiber prediction models were built using the modified partial least squares regression (MPLS) and eight different mathematical treatments (1,4,4,1; 1,5,5,1; 1,8,8,1; 1,10,10,1; 2,4,4,1; 2,5,5,1; 2,8,8,1; and 2,10,10,1; where the first digit is the derivative, the second is the gap between the data points, the third indicates the first smoothing data points, and the fourth the second smoothing data points) were tested. These pre-treatments have been previously successfully applied in samples having similar spectral characteristics [36, 50].

The performance of the models was evaluated by means of the following statistics: minimum standard error of calibration (SEC), minimum standard error of prediction (SEP), greatest coefficient of determination for calibration (R^2^_CAL_), greatest coefficient of determination for validation (R^2^_VAL_), the ratio of performance to deviation (RPD, *i*.*e*., the ratio of reference standard deviation with SEP), and the range error ratio (RER, *i*.*e*., the ratio between the range of the reference data and the SEP). Based on literature, good predictions should have an RPD ≥ 3.0 and/or RER > 10 [52, 53]. However, due to the specificities of sample preparation (*e*.*g*., soils, feces, feeds, forages), some adjustments have been made to the RPD thresholds. Here, we considered RPD ≥ 3 an accurate calibration, 2 ≥ RPD > 3 a calibration suitable for screening, and RPD < 2 was considered as a poor calibration [54].

Eighty-two fecal samples from three herbivore species with different digestive physiologies (*i*.*e*. 60 red deer, 11 cattle and 11 horses) were used to develop the three fecal fiber models with and without the addition of sodium sulfite in NDF laboratory determination. Subsequently, 445 fecal samples from six herbivore species (Alpine ibex, red deer, sheep, Alpine chamois, Pyrenean chamois, and Italian hare) were used to develop the fiber multispecies models. A subset of 357 samples (80% of the total samples) formed the calibration set, and 88 samples (20% of the total samples) were previously seared and used as cross-validation set (Table 2). For each species, the sample subsets were randomly selected. To assess the predictive accuracy of the multispecies equations, 63 samples from five herbivore species not included previously, were used as external validation set (Table 2).

**Table 2:** Database of the multispecies calibration and validation sets.

Species	Total	Calibration	Validation	Species	Validation
**Multispecies calibration**				**External validation**	
Alpine ibex	100	80	20	European mouflon	20
Red deer	100	80	20	Cattle	11
Sheep	100	80	20	Horse	11
Italian hare	72	58	14	Roe deer	11
Pyrenean chamois	48	38	10	European rabbit	10
Alpine chamois	30	26	4		
**TOTAL**	**445**	**357**	**88**	**TOTAL**	**63**

We conducted a sensitivity analysis [55] to determine the robustness of our NIRS predictions across species following Cinelli and Hazlett [56] and the “sensemakr” R package version 0.1.4 [57]. This analysis included the estimation of the partial R^2^ of treatment with outcome, *i*.*e*., the proportion of the variance of NIR predictions explained by laboratory determinations after accounting for the species covariate and the RV (robustness value) that quantifies how much an unobserved confounding covariate would be needed to change the estimated treatment effect to zero. The linear model estimates and associated statistics (S.E., t-value, p-value) for the laboratory-NIR analysis were also calculated.

## Results

**Fig 2:**
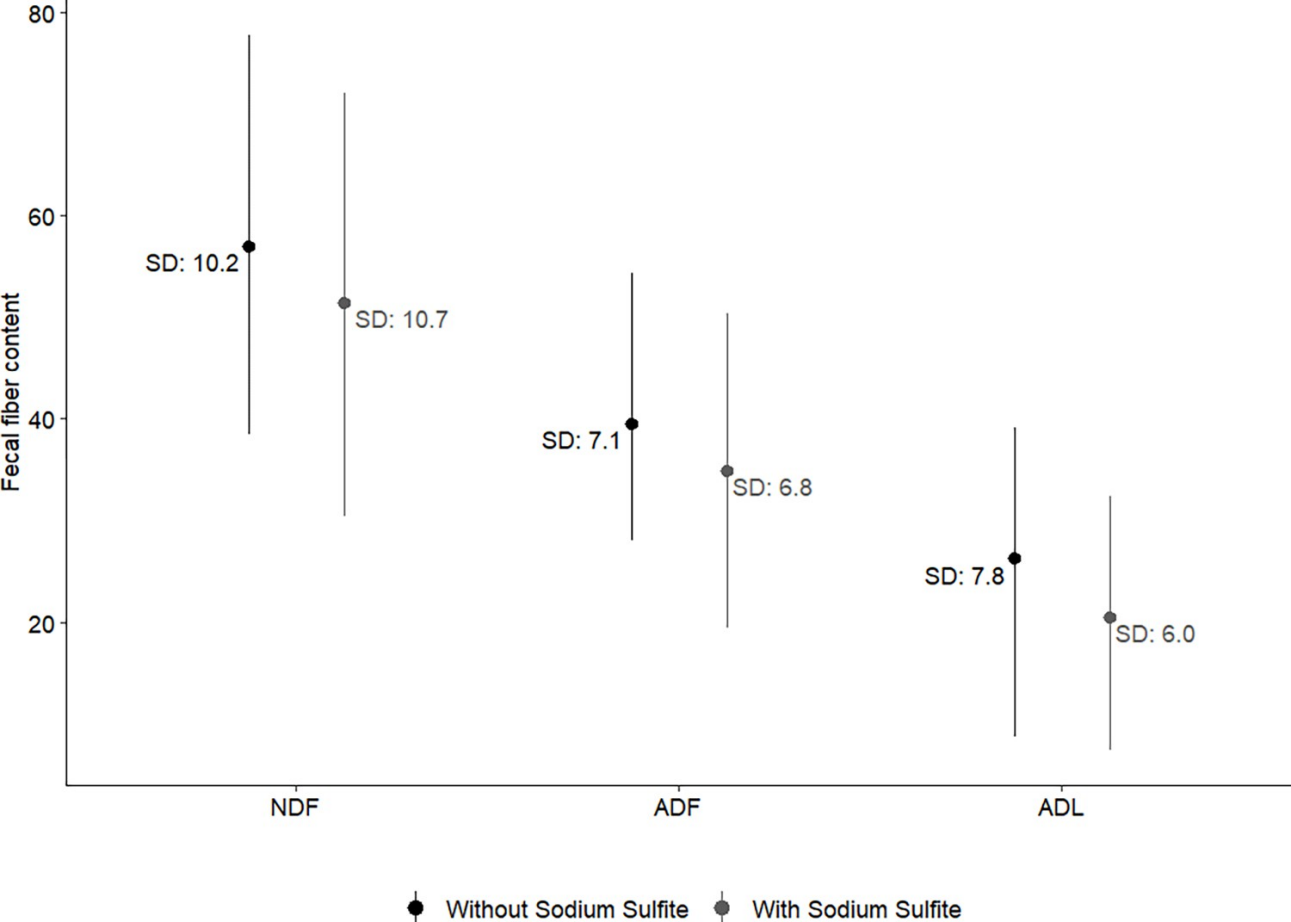
Fiber composition of herbivore fecal samples (neutral detergent fiber NDF, acid detergent fiber ADF, and acid detergent lignin ADL) used in the NIRS analysis. Fiber fractions were determined with and without addition of sodium sulfite (results are expressed in % dry matter).

The addition of sodium sulfite during the NDF analysis reduced the NDF, ADF and ADL values (NDF, r = 0.87, P < 0.0001; ADF, r = 0.81, P < 0.0001; ADL, r = 0.70, P < 0.0001, Fig 2), and differences for the matched pairs were normally distributed. The optimal spectral pre-treatments (*i*.*e*., math treatment and scatter correction) were selected and are shown in Table 3. The results of calibration and cross-validation were similar for both models (with/without sodium sulfite), with better predictive power for NDF and ADF (R^2^_CAL_ ≥ 0.97 and R^2^_CV_ ≥ 0.90) than for ADL (R^2^_CAL_ ≥ 0.94 and R^2^_CV_ ≥ 0.89; Table 3). In general, the predictive power was slightly greater without sodium sulfite than with sodium sulfite (RPD 3.7 to 5.4 vs. 3.1 to 4.2). Thus, the multispecies models were developed without using sodium sulfite during the laboratory procedure.

**Table 3:** Statistics of calibration and cross-validation of predictive models used for determination of fiber contents in fecal samples by NIRS analysis. Fiber fractions were determined with and without the addition of sodium sulfite.

	^a^Math treatment	^b^Scatter correction	without sodium sulfite					with sodium sulfite				
			R^2^_CAL_	SEC	R^2^_CV_	SECV	RPD	R^2^_CAL_	SEC	R^2^_CV_	SECV	RPD
NDF	1,4,4,1	SNV+D	0.97	1.91	0.94	2.42	4.2	0.97	1.93	0.94	2.55	4.2
ADF	2,4,4,1	SNV+D	0.97	1.30	0.90	1.30	5.4	0.97	1.23	0.91	2.05	3.3
ADL	1,4,4,1	MSC	0.96	1.67	0.93	2.09	3.7	0.94	1.47	0.89	1.92	3.1

^a^ Math treatment: derivative order, subtraction gap, first smoothing, second smoothing.

^b^ SNV—standard normal variate, D—detrend, MSC—multiple scatter correction. R^2^CAL—coefficient of determination for calibration; SEC—standard error of calibration; R^2^_CV_—coefficient of determination for cross-validation; SECV—standard error of cross-validation; RPD—ratio of performance to deviation (SD/SECV); NDF—neutral detergent fiber; ADF—acid detergent fiber; ADL—acid detergent lignin.

The compositional data of the herbivore fecal samples presented a wide range of values in both the calibration and validation matrices for each fiber (Table 4). Furthermore, the validation range for each fiber was within the range of the calibration for that same fiber [58]. The best spectral model (*i*.*e*., best math treatment and best scatter correction) for each fiber is represented in Table 5. The calibration and cross-validation results showed high predictive power for NDF, ADF and ADL determination (R^2^_CAL_ ≥ 0.93 and R^2^_VAL_ ≥ 0.91; Table 5 and S1 Fig). The RPD values ranged from 3.4 to 3.9 and RER values ranged from 14.2 to 19.4 (Table 5). Both statistics showed that the three models were accurately calibrated [52]. Moreover, better calibrations (*i*.*e*., higher RPD values) [53] were obtained when predicting NDF values, while poorer calibrations were obtained when predicting ADL values. The standard error of calibration (SEC) and standard error of prediction (SEP) were also lower than 3 for all three fiber fractions.

**Table 4:** Summary of fiber fractions (% of dry matter) from herbivore fecal samples used in the multispecies calibration and validation datasets.

		Calibration set				Validation set			
		N	Range	Mean	SD	N	Range	Mean	SD
Multispecies	NDF	357	19.7–81.4	52.7	9.9	88	25.8–80.1	55.3	11.0
	ADF	357	13.0–62.8	34.4	7.9	88	16.4–56.5	36.5	8.2
	ADL	357	2.3–40.4	12.7	6.3	88	3.1–30.4	13.5	6.6
External validation set (other species)									
Species		Range	Mean	SD	Species		Range	Mean	SD
E. rabbit	NDF	40.9–64.4	55.1	7.6	Cattle	NDF	47.1–74.7	65.5	10.3
	ADF	26.5–40.3	34.8	4.5		ADF	33.7–54.3	46.0	7.9
	ADL	4.4–12.0	8.0	2.0		ADL	11.2–26.8	18.5	5.6
E. mouflon	NDF	34.8–64.3	48.7	9.4	Horse	NDF	54.5–77.7	67.6	8.3
	ADF	8.0–52.2	33.0	10.7		ADF	32.0–51.4	45.0	5.9
	ADL	9.7–36.0	17.2	6.4		ADL	8.7–20.1	13.8	3.1
Roe deer	NDF	49.0–71.4	62.7	6.8					
	ADF	37.0–56.1	49.1	5.8					
	ADL	22.3–31.9	29.6	3.0					

Fiber content was determined without the addition of sodium sulfite.

N—number of samples; Range—interval between the maximum and minimum value of data set; SD—standard deviation; NDF—neutral detergent fiber; ADF—acid detergent fiber; ADL—acid detergent lignin.

**Table 5:** Statistics of calibration and validation of predictive models used for determination of fiber content in herbivore fecal samples by NIRS analysis.

	Calibration				Validation					
	^a^Math treatment	^b^Scatter correction	R^2^_CAL_	SEC	R^2^_VAL_	SEP	Bias	Slope	RPD	RER
NDF	1,4,4,1	SNV	0.94	2.42	0.93	2.80	0.41	1.04	3.9	19.4
ADF	1,5,5,1	SNV+D	0.93	2.03	0.91	2.44	0.01	0.99	3.4	16.4
ADL	2,4,4,1	MSC	0.96	1.25	0.91	1.92	0.06	0.99	3.4	14.2

^a^ Math treatment: derivative order, subtraction gap, first smoothing, second smoothing.

^b^ SNV—standard normal variate, D—detrend, MSC—multiple scatter correction. R^2^CAL—coefficient of determination for calibration; SEC—standard error of calibration; R^2^_VAL_—coefficient of determination for validation; SEP—standard error of prediction; RPD—ratio of performance to deviation (SD/SEP); RER—range error ratio (Range/SEP); NDF—neutral detergent fiber; ADF—acid detergent fiber; ADL—acid detergent lignin.

Our sensitivity analysis for evaluating the robustness of the relationship between laboratory determination and NIR prediction of fecal NDF, ADF and ADL, showed high values for the partial R^2^ (91.02% for NDF, 90.73% for ADF and 90.72% for ADL, Table 6), suggesting that any confounder would not have a significant influence on the ability of NIRS to predict fecal NDF, ADF and ADL contents across mammal species. Along the same lines, unobserved confounders would need to explain more than 91% for NDF, 90% for ADF, and 91% for ADL to nullify the NIRS predictions for the fecal fiber contents (Table 6).

**Table 6:** Summary of the linear regressions exploring the relationships between NIRS predictions and laboratory determinations of NDF, ADF and ADL concentrations in 333 fecal samples of red deer (*Cervus elaphus*, N = 75), chamois (*Rupicapra* sp, N = 48), sheep (*Ovis aries*, N = 78), Alpine ibex (*Capra ibex*, N = 77) and Italian hares (*Lepus corsicanus*, N = 55).

Fixed Factor	Est	S.E.	t-value	R^2^_outcome_ (%)		p-value	RV _q = 1, α = 0.05_
NDF	0.92	0.01	57.58		91.02	2.01e-16	0.91
ADF	0.92	0.02	55.71		90.73	2.00e-16	0.90
ADL	0.93	0.01	55.61		90.72	2.00e-16	0.91

R^2^_outcome_—the proportion of the variance of NIR predictions explained by laboratory determinations after accounting for the species covariate; RV _q = 1, α = 0.05_—the robustness value calculated with q = 1 and α = 0.05, this represents the proportion of the remaining variance that unobserved confounders would need to explain to nullify the observed association at the 95% confidence interval; NDF—neutral detergent fiber; ADF—acid detergent fiber; ADL—acid detergent lignin.

**Fig 3:**
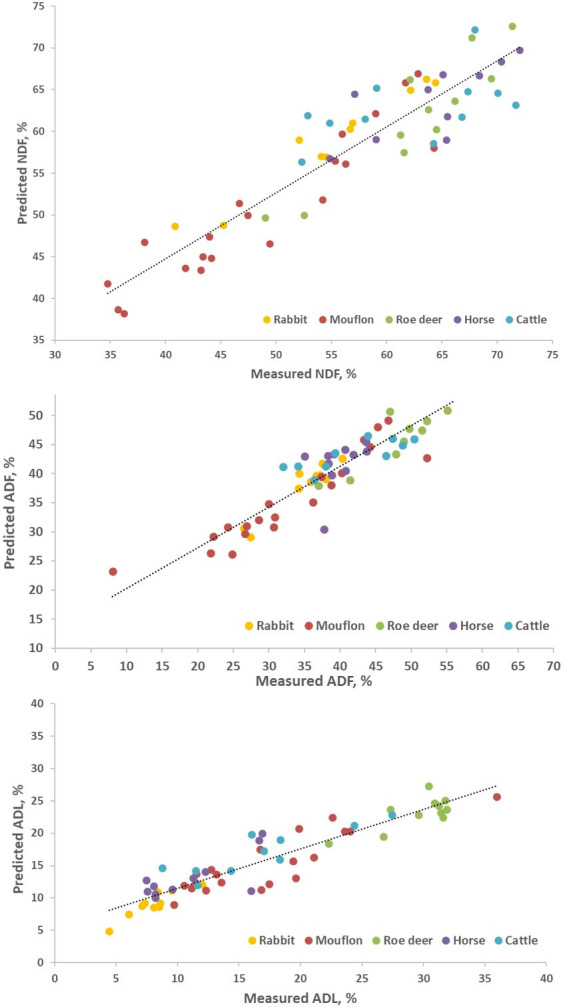
Relationship of fiber fractions (neutral detergent fiber NDF, acid detergent fiber ADF, and acid detergent lignin ADL) values of fecal samples used as external validation versus NIRS values predicted with the multispecies models.

The results of the predictions for fecal fiber contents in the species not used for the calibration showed an R^2^_VAL_ between 0.66 and 0.95, with consistently better values for NDF and ADF than for ADL (Table 7). The RPD values ranged between 0.5 and 3.2, with the European mouflon presenting the best prediction values, and the European rabbit and cattle the poorest. Roe deer and horse showed a lower RPD value for ADL prediction. The relationship between reference fiber values (NDF, ADF and ADL) of the species used as external validation and the predicted values by NIRS multispecies model is represented in Fig 3.

**Table 7:** Validation statistics using other herbivore species to predict the fiber content (% of dry matter) in fecal samples with the multispecies models.

Species		R^2^_VAL_	SEP	Bias	Slope	RPD	Species		R^2^_VAL_	SEP	Bias	Slope	RPD
E. rabbit	NDF	0.94	3.68	-3.39	1.08	2.1	Cattle	NDF	0.87	5.00	2.15	1.39	2.1
	ADF	0.95	2.97	-2.81	1.00	1.5		ADF	0.86	4.82	3.74	1.22	1.6
	ADL	0.89	1.17	-0.95	0.98	1.7		ADL	0.81	3.24	2.31	1.35	1.7
E. mouflon	NDF	0.86	3.16	-0.29	1.02	3.0	Horse	NDF	0.89	7.08	6.47	1.14	2.3
	ADF	0.85	3.73	-1.82	1.16	2.9		ADF	0.91	5.89	5.65	0.96	2.1
	ADL	0.81	2.01	-0.02	1.06	3.2		ADL	0.86	1.56	0.99	1.03	1.2
Roe deer	NDF	0.90	2.77	1.40	0.87	2.5							
	ADF	0.80	2.84	0.78	0.84	2.0							
	ADL	0.66	6.13	5.89	0.92	0.5							

R^2^_VAL_—coefficient of determination for validation; SEP—standard error of prediction; RPD—ratio of performance to deviation (SD/SEP); NDF—neutral detergent fiber; ADF—acid detergent fiber; ADL—acid detergent lignin.

## Discussion

Recently, NIRS multispecies models have demonstrated their usefulness in the study of animal nutritional ecology, and consequently in the conservation and management of herbivore species [59]. Some studies have proven the effectiveness of NIRS multispecies models using fecal samples [36, 48], and a study of fiber contents in rumen and fecal samples [29] suggests a possible applicability of NIRS to fiber studies using fecal samples of several species [60]. This study demonstrated for the first time that NIRS multispecies models can be used to assess the fiber content in feces of a broad-spectrum of herbivore species. Furthermore, we concluded that sodium sulfite does not contribute to improve NDF calibration and, therefore, our multispecies models can be developed without this compound.

Sample treatment with sodium sulfite is recommended for samples with high nitrogen content (*e*.*g*., feeds) [23], however, its use has been considered optional due to its impact on fibrous compounds [14]. In this study, the use of sodium sulfite in NDF analysis during pre-calibration reduced the values of NDF, ADF and ADL contents. This observation is well documented in the literature [24, 61]. Gomes and colleagues [24] have observed that sodium sulfite increased precision while lowering accuracy on fiber compounds estimates in tropical forages, consequently advised against its use. In fact, some authors have already performed this analysis without including this reagent [62, 63]. This could cause some constraints to the accurate determination of fiber content since protein contamination is still present. However, the protein load is considerably low in fecal samples as it consists of indigestible nitrogenous matter [64]. Moreover, in this study, calibration and cross-validation statistics have shown that the results were better in the absence of sodium sulfite.

The development of NIRS models requires diverse datasets to effectively capture all sources of variation and ensure accurate predictions [26]. Here, herbivore species included in the calibration dataset have different digestive physiologies and diet compositions. Furthermore, the diversity of the fecal samples in this study also accounted for other potential sources of variability in fiber values, such as season [2, 65] and types of diet [20]. This contributed to a relatively broad spectrum of values for the three fiber fractions in both the calibration and validation matrices, as recommended for accurate NIRS models [58]. In this study, the values for fecal NDF, ADF and ADL content agreed with previously published values [33, 66–69], covering the wide ranges reported for herbivores with different types of diet (*i*.*e*., grazers, browsers and intermediate feeders) [70].

The robust calibration values obtained for NDF, ADF and ADL determination were similar to previous species-specific NIRS equations [66, 69]. The validation R^2^_VAL_ and RPD values (Table 7) demonstrated the high predictive value of the obtained equation. The values from the multispecies equations were similar [35, 66] or even better [33, 67, 68] than previously published species-specific equations for fiber content determination. Thus, this multispecies equation predicts better the fecal fiber content than previous monospecies equations. Calibration and validation statistics were in agreement with other multispecies equations to assess other nutritional indicators in herbivore feces [36, 48] and fiber content on grasses [71].

External validation is recommended when the aim is to expand the use of the equation obtained for different situations [72]. This is done by predicting samples with some degree of variability from the calibration set, such as different species, seasons, and/or environmental contexts, as was the case for this study. The R^2^_VAL_ above 0.8 of the validation with other species (Table 7) can be considered satisfactory [20], except for the ADL contents in roe deer feces (R^2^_VAL_ = 0.66). The RPD values were also generally satisfactory, considering that the equations produced were suitable for screening [54], except for ADF and ADL values for rabbit and cattle and ADL values for roe deer and horse (Table 7). Mouflon was the best-predicted external species (higher RPD values), probably due to their flexible diet with low seasonal variability [73], especially in Mediterranean environments [74]. Conversely, the species with the worst predicted fiber content were cattle and rabbits, probably due to the particularities of their digestive systems. Cattle, as a foregut fermenter, has a digestive system and microorganisms that are highly specialized and more efficient than other ruminants in digesting fibers [75]. Whereas for rabbits, coprophagy is a confounding factor, since the reingestion and re-digestion of feces may cause variability in the fecal fiber content. Hares, the most similar herbivore used in the calibration dataset, have a lower hemicellulose digestibility and lower coprophagy rate than rabbits [76], resulting in differences in fecal fiber contents (*e*.*g*., NDF values [77]). When considering ADL calibrations, the poorest validations were observed for roe deer and horse. Both species are at opposite extremes in terms of trophic ecology, since the roe deer is a browser and the horse a grazer. Thus, we expect higher ADL content from the former and lower for the latter [70], as shown in Table 5. However, both species can adapt their diet depending on the availability of resources [78, 79]. Hence, fecal fiber content variation on roe deer and horse may depend on location, season and year. Therefore, the specific differences in i) the digestive system of cattle and rabbits and ii) the diet preferences of roe deer and horses, may result in increased difficulty in predicting fiber values in feces using an equation obtained with other herbivores.

In general, the equations for predicting NDF values performed better than the ADF equations, which in turn were better than the ADL equations. Such decreasing trend was also observed in the initial calibration with and without sodium sulfite and is supported by other studies [27, 32, 33, 67, 68]. This occurs because fiber determination using the Van Soest [14] method is a sequential protocol, starting with NDF and ending with ADL, which leads to cumulative errors. The laboratory error increases with more handling steps since they decrease the concentration of the trait used for prediction, and, therefore, influence the accuracy of the prediction models [80]. In addition to these methodological nuances, there are also errors inherent to the NIRS technique (see [42]). Nevertheless, this technology has been effective compared to conventional chemistry [26].

The variability of the samples used (*i*.*e*., species with different digestive physiologies and diet preferences) to calibrate NIRS models is a condition often mentioned as crucial to the applicability of the models [80]. However, species variability in this study may be a constraint as it may correspond to confounding factors (*e*.*g*., differences in diet composition across species) and compromise accurate fiber prediction for all species. Here, the sensitivity analysis showed that these unmeasured confounding factors (*i*.*e*., species) did not influence the results. Thus, the relationship between the laboratory values and the values predicted by NIRS is so robust that the variability of digestive physiology and diet does not affect the results. Moreover, this analysis reinforced the applicability of this multispecies model in the future. Nonetheless, continuous validation is necessary to monitor the accuracy and precision of the calibration equations [37]. However, caution is needed when applying this model to predict fecal fibers from herbivores with very distinct diets (*e*.*g*., consumption of plant species with high tannin content, [81]). In this case, it is recommended to analyze some samples in the laboratory and compare them with the values predicted by the model [82]. After checking that the model is still robust, the remaining fecal fiber values for this new species can be predicted.

Despite some exceptions marked by low RPD values, the multispecies models could be used for screening NDF, ADF, and ADL contents in the feces of various herbivore species. Low RPD values during external validation could be overcome by increasing the variability and broadness of samples used in the calibration dataset. In fact, the main challenge of using fecal NIRS to monitor animal nutrition is to obtain a calibration dataset with adequate variability [27]. Coates and Dixon [83] produced a calibration to estimate non-grass proportion in cattle diet that has been developed, validated, and continuously improved through the addition of fecal samples over the years. Thus, future efforts to increase the number of herbivore species used in the initial dataset to fit the multispecies models, as well as to include a high amount of spatial and temporal variability within the dataset, would improve and refine the first calibrations carried out in this study. Specifically, future research should focus on adding samples of herbivore species with highly specific digestive systems or extreme dietary preferences, living in highly seasonal environments. This can be accomplished more efficiently by relying on collaborations between research groups to interchange samples and data [26], as performed in the present study.

## Conclusions

The NIRS multispecies equations developed in this study represent a significant advancement in fecal fiber prediction, as they are the first to accurately predict fecal fiber content across multiple herbivore species. Unlike previous species-specific models, these equations can reliably estimate fecal fiber content for species not included in the original dataset. This innovation eliminates the need for species-specific calibration and validation, facilitating nutritional ecology assessments even with limited fecal samples–a crucial advantage for studying endangered or hard-to-reach populations. However, to enhance their accuracy and applicability, these models must be continuously updated with data encompassing diverse species, diets, seasons, locations, and management practices.

Given that nutritional condition is a key driver of individual fitness, this work marks an important methodological advance in understanding population dynamics. The multispecies NIRS models offer a valuable tool for conservation and management, enabling efficient monitoring of feeding behavior and nutritional ecology in herbivore species.

## Supporting information

S1 Fig Linear relationship between NIRS predicted data and chemical reference data for (A) neutral detergent fiber (NDF), (B) acid detergent fiber (ADF) and (C) acid detergent lignin (ADL), in herbivore fecal samples for the calibration set. R^2^_CAL_—coefficient of determination for calibration; SEC—standard error for calibration.
(PDF)
