# Supplementary material for: Predicting fiber content in herbivore fecal samples using a multispecies NIRS model
Source: PLoS One. 2025 Jan 8;20(1):e0317145. doi: 10.1371/journal.pone.0317145 (PMC11709307; doi:10.1371/journal.pone.0317145)
Supplement: S1 Fig — Linear relationship between NIRS predicted data and chemical reference data for (A) neutral detergent fiber (NDF), (B) acid detergent fiber (ADF) and (C) acid detergent lignin (ADL), in herbivore fecal samples for the calibration set. R2CAL—coefficient of determination for calibration; SEC—standard error for calibration. (PDF) [file pone.0317145.s001.pdf]

S1 Appendix

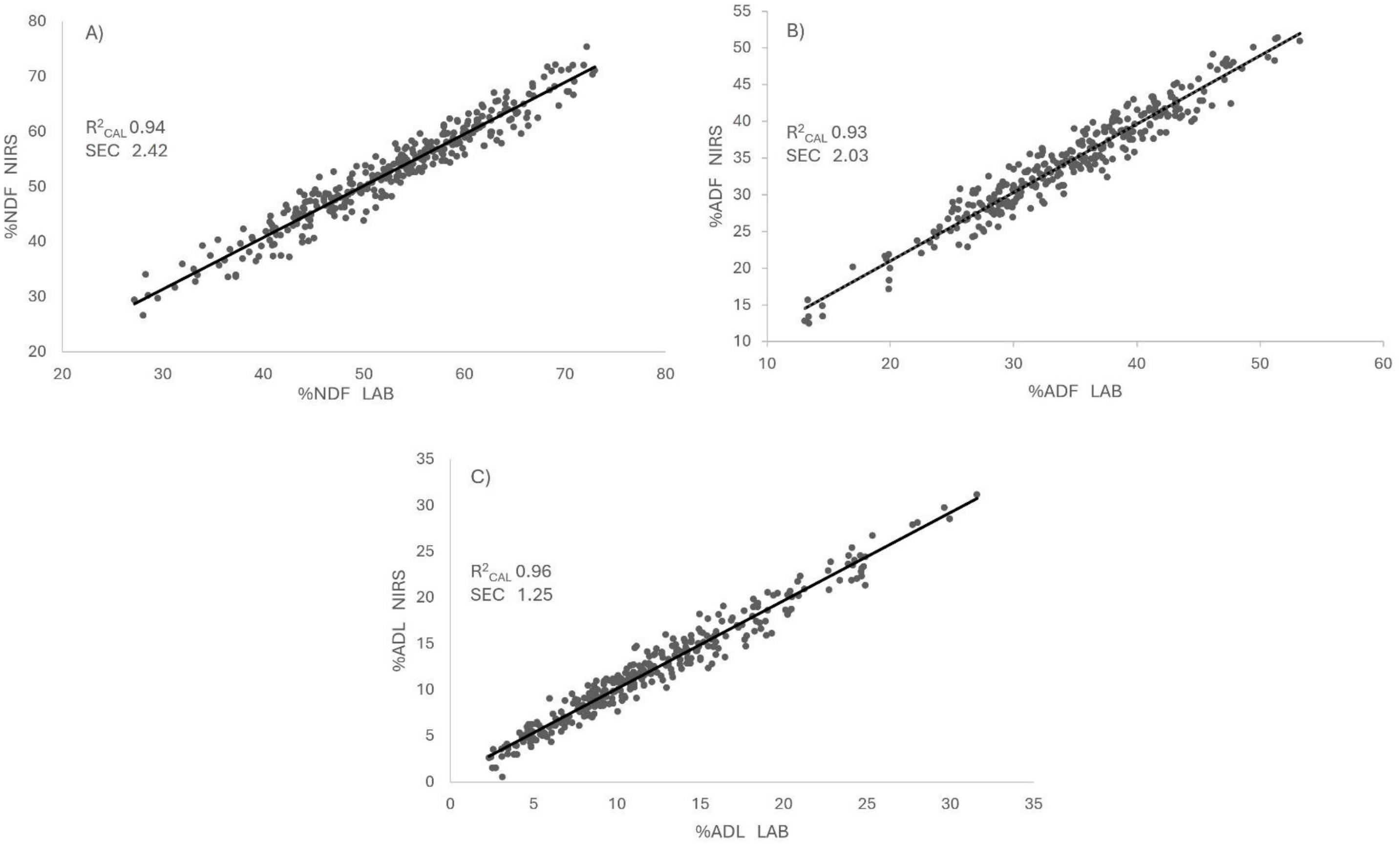

Figure S1 - Linear relationship between NIRS predicted data and chemical reference data for (A) neutral detergent fiber (NDF), (B) acid detergent fiber (ADF) and (C) acid detergent lignin (ADL), in herbivore fecal samples for the calibration set. R^2_{CAL} - coefficient of determination for calibration; SEC - standard error for calibration.
